# Treatment of acute myocardial infarction complicated by ventricular septal rupture with delayed surgery following intra-aortic balloon pump support: A case report

**DOI:** 10.1097/MD.0000000000046911

**Published:** 2026-01-09

**Authors:** Shuang Guo, Xuping Cheng, Xuandong Jiang

**Affiliations:** a Intensive Care Unit, Affiliated Dongyang Hospital of Wenzhou Medical University, Dongyang, Zhejiang, PR China.

**Keywords:** acute myocardial infarction, delayed surgery, intra-aortic balloon pump, ventricular septal rupture

## Abstract

**Rationale:**

Inferior wall myocardial infarction (MI) complicated by ventricular septal rupture (VSR) is a rare but fatal complication of acute MI. Early surgical intervention is associated with a high mortality rate, and the optimal timing for surgery remains uncertain. This paper presents a case of surgical management of acute inferior wall MI complicated by VSR, with the aim of exploring diagnostic and therapeutic strategies, as well as the timing of surgical intervention.

**Patient concerns:**

A 74-year-old man was admitted to our hospital with chest tightness and tachypnea lasting 3 days. He had no history of hypertension, diabetes mellitus, or hyperlipidemia, denied a family history of coronary heart disease, and had a smoking history of over 30 years.

**Diagnoses:**

Coronary angiography revealed occlusion in the proximal to mid-segment of the right coronary artery, and echocardiography indicated VSR.

**Interventions:**

Surgical treatment was performed after 20 days of intra-aortic balloon pump counterpulsation support.

**Outcomes:**

The patient’s condition improved postoperatively, and he was eventually discharged.

**Lessons:**

VSR is a rare complication of acute MI. Although early surgical intervention is generally preferred, the optimal timing remains ambiguous, and delaying VSR repair may be advantageous. In hemodynamically unstable patients, the use of temporary mechanical circulatory support devices to postpone surgical intervention is advisable.

## 1. Introduction

Ventricular septal rupture (VSR) is a rare but fatal complication of acute myocardial infarction (AMI). The incidence of ST-segment elevation myocardial infarction (STEMI) is higher than that of non-STEMI.^[1]^ Medication therapy alone presents a significantly high risk of early mortality, and surgical intervention remains the preferred treatment. Nonetheless, surgery is also associated with elevated mortality rates, and the optimal timing for intervention remains uncertain.^[1,2]^ We present a case of acute inferior wall myocardial infarction (MI) complicated by VSR, managed with delayed surgical intervention following intra-aortic balloon pump (IABP) support, which resulted in favorable postoperative recovery. The case details are reviewed alongside relevant literature to explore diagnostic and therapeutic strategies, as well as optimal surgical timing for VSR.

## 2. Case presentation

**Figure 1.**
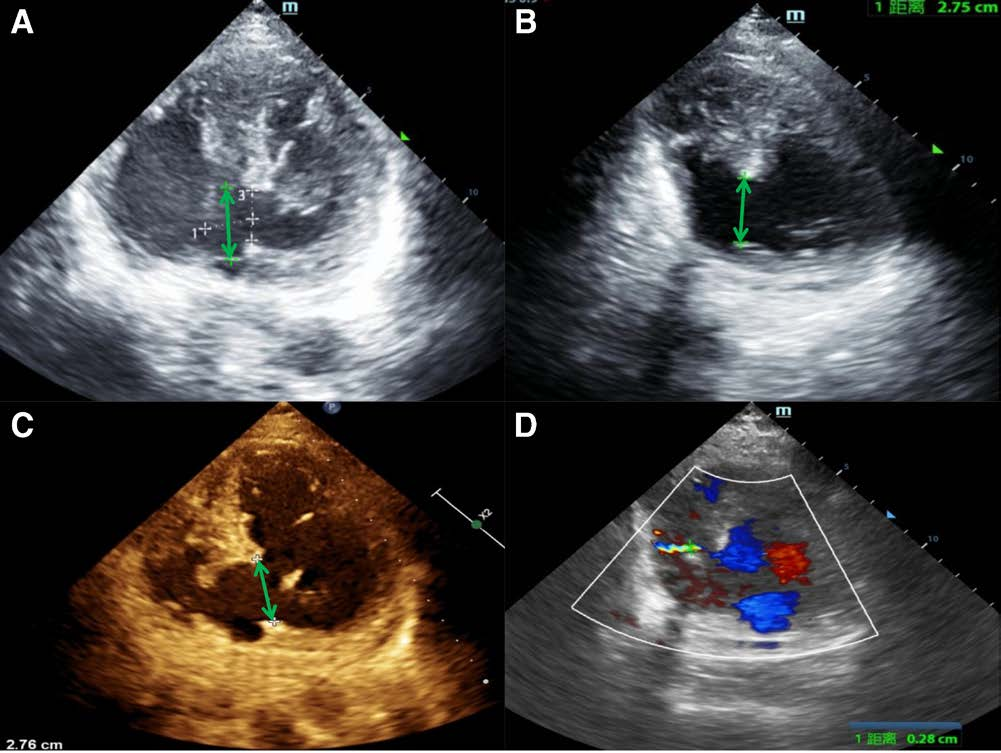
Ultrasound images on (A) day 0: a 17.1-mm defect in the ventricular septum; (B) day 10: the ventricular septal defect widened to approximately 27.5 mm at the left ventricular aspect; (C) day 17: the defect measured approximately 27.6 mm at the left ventricular aspect; and (D) day 27: postoperative image showing a residual defect at the inferior margin of the patch, measuring approximately 2.8 mm at the right ventricular aspect.

A 74-year-old man was admitted to our hospital with chest tightness and tachypnea persisting for 3 days. He had no history of hypertension, diabetes mellitus, or hyperlipidemia; denied a family history of coronary heart disease; and had a smoking history of over 30 years. Three days before admission, he experienced paroxysmal chest tightness and tachypnea triggered by physical activity, which was alleviated by 1 to 2 minutes of rest. Additionally, the patient reported abdominal distension, nausea, and vomiting. Laboratory tests revealed a high-sensitivity troponin T level of 1.04 ng/mL and an N-terminal pro-B-type natriuretic peptide level of 17,393.0 pg/mL. Electrocardiography showed sinus rhythm, Q-wave formation in leads III and aVF, and ST-T changes in leads V1–V4. Color Doppler echocardiography indicated a ventricular septal defect (VSD), aneurysm formation in the membranous septum, and a left-to-right shunt at the ventricular level, along with degenerative changes in the aortic valve with mild regurgitation, mild mitral regurgitation, mild-to-moderate tricuspid regurgitation, moderate-to-severe pulmonary arterial hypertension, right atrial enlargement, and aortic atherosclerosis (Fig. 1), with an ejection fraction of 71%. Physical examination revealed a pulse rate of 83 beats/min, respiratory rate of 20 breaths/min, blood pressure of 110/74 mm Hg, and body temperature of 36.5°C. The patient was conscious and alert, showed no jugular venous distention, and had moist rales in both lungs. Cardiac examination revealed no obvious enlargement, a heart rate of 83 beats/min with a regular rhythm, and systolic murmurs audible in all valve areas, most prominently at the third to fourth intercostal space along the left sternal border. The abdomen was soft, with no tenderness or rebound tenderness. Neither the liver nor the spleen was palpable below the costal margin, and no abdominal vascular murmurs were detected. Both lower limbs showed no edema. The following diagnoses were made: acute STEMI, Killip class III; coronary atherosclerotic heart disease; post-AMI left-to-right shunt (VSR); moderate-to-severe pulmonary hypertension; and membranous ventricular septal aneurysm.

**Figure 2.**
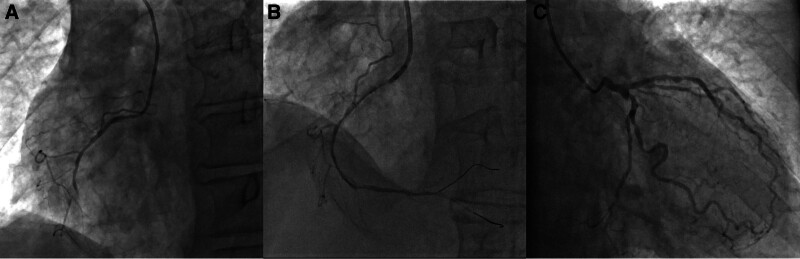
Angiography images of (A) right coronary artery showing occlusion; (B) right coronary artery after balloon angioplasty (PTCA); and (C) left coronary artery. PTCA = percutaneous transluminal coronary angioplasty.

Emergency coronary angiography revealed the following findings: 20% stenosis with calcification in the left main coronary artery; 50% stenosis in the proximal segment and 60% in the distal segment of the circumflex artery, both with thrombolysis in MI grade 3 distal blood flow; and diffuse stenosis in the proximal to mid-segment of the left anterior descending artery, with maximum stenosis of 70% and thrombolysis in MI grade 3 distal blood flow; and diffuse stenosis in the proximal to mid-segment of the right coronary artery, with maximum stenosis of 75% and occlusion beyond the mid-segment. A percutaneous transluminal coronary angioplasty was performed on the right coronary artery (Fig. 2).

**Figure 3.**
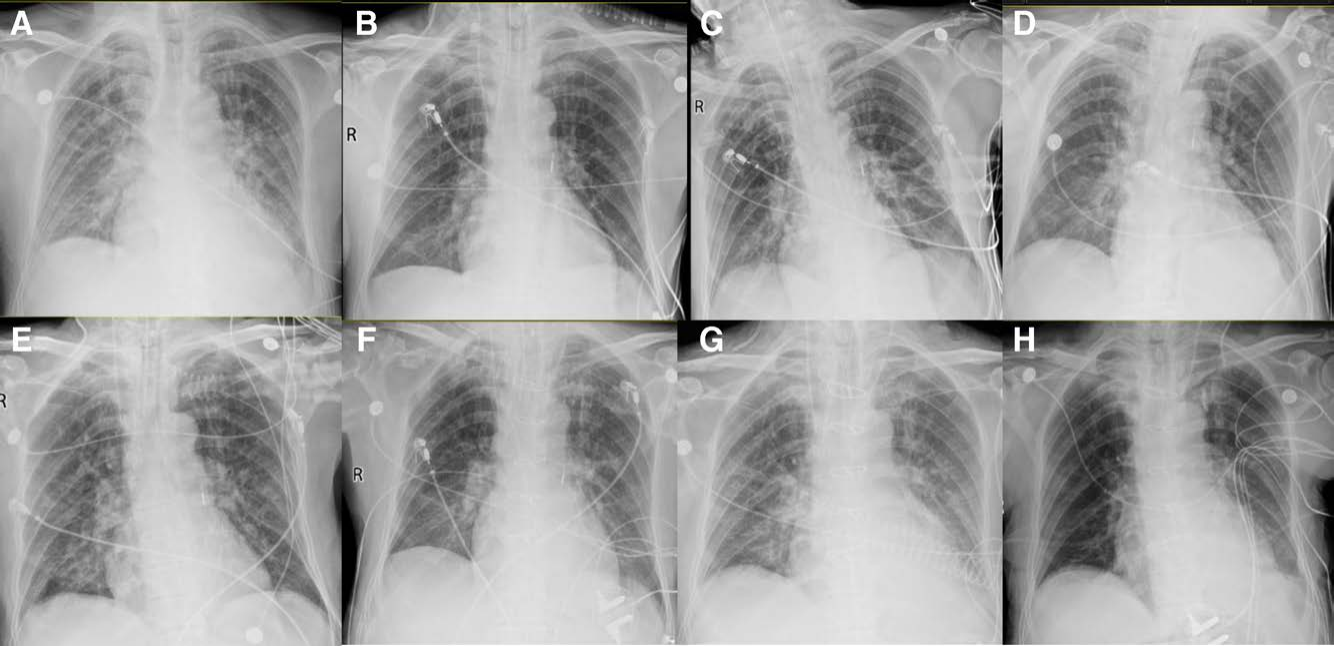
Chest x-ray images on (A) day 0: preintubation period following ICU admission; (B) day 6; (C) day 10; (D) day 16; (E) day 19: 24 hours preoperatively; (F) day 20: within 24 hours postoperatively; (G) day 22: the day following IABP discontinuation; and (H) day 28. IABP = intra-aortic balloon pump, ICU = intensive care unit.

Postoperatively, the patient was transferred to the intensive care unit for further monitoring. On day 0, he developed tachypnea and hypotension. Chest radiography revealed pulmonary edema (Fig. 3). The patient underwent endotracheal intubation with mechanical ventilation for respiratory support, along with IABP counterpulsation. A norepinephrine infusion (0.06–0.12 µg/kg/min) was initiated to stabilize blood pressure, and the following medications were administered: enteric-coated aspirin 100 mg orally once daily, weight-based heparin sodium (to maintain an activated partial thromboplastin time between 55 and 74 seconds), and atorvastatin calcium 20 mg orally at bedtime. Given the high bleeding risk associated with dual antiplatelet therapy and the potential need for imminent cardiac surgery if the patient’s condition deteriorated, we opted for a regimen of aspirin monotherapy combined with unfractionated heparin anticoagulation. Follow-up echocardiography revealed enlargement of the VSR (Fig. 1) and persistent pulmonary exudation (Fig. 3). Extubation proved challenging, and the patient developed fever and secondary pulmonary infection, requiring intravenous administration of piperacillin sodium/tazobactam sodium 4.50 g every 8 hours. After the pulmonary infection was effectively managed, the patient underwent coronary artery bypass grafting with cardiopulmonary bypass, VSD repair, and temporary pacemaker implantation under general anesthesia on day 20. An irregular defect measuring approximately 3 cm× 2 cm was identified in the basal portion of the posterior ventricular septum. On day 21, IABP support and heparin sodium were discontinued, and clopidogrel bisulfate 75 mg was administered orally once daily. The patient was extubated on day 22. Postoperative echocardiography performed on day 27 revealed a residual shunt (Fig. 1). Secondary surgery was not performed owing to the small shunt volume. The patient was transferred to the general ward on day 29 and discharged on day 45. After discharge, the patient continued to receive enteric-coated aspirin 100 mg orally once daily, clopidogrel bisulfate 75 mg orally once daily, and atorvastatin calcium 20 mg orally at bedtime. At the 1-month follow-up, the patient was able to manage daily activities independently and walk outdoors.

## 3. Discussion

This case prompted a thorough examination of diagnostic and treatment strategies for VSR. Our patient recovered favorably following the outlined treatments. VSR is the most prevalent mechanical complication post-AMI, with an incidence of approximately 0.21% in patients with STEMI and 0.04% in those with non-STEMI.^[1]^ Risk factors for VSR following AMI include advanced age, female sex, delayed reperfusion therapy, chronic kidney disease, a high Global Registry of Acute Coronary Events score, and acute STEMI.^[3]^ VSR typically occurs within the first week after AMI, exhibiting a bimodal distribution: the first peak occurs within 24 hours of infarction, and the second peak arises approximately 5 days later. However, with primary percutaneous coronary intervention now being the standard treatment, there is a noted trend toward earlier occurrences of VSR, primarily within the first day of MI.^[4]^ Transthoracic echocardiography serves as a rapid, sensitive, and noninvasive method for diagnosing VSR, and right heart catheterization remains the gold standard for diagnosing post-MI VSR.^[5]^

The location and type of VSR depend on the coronary artery affected by the transmural MI. Ischemic VSDs in the anterior wall and apex are typically associated with infarction in the territory supplied by the left anterior descending artery, whereas posterior wall VSDs result from inferior wall MI. Posterior VSDs are often accompanied by mitral regurgitation and usually stem from the ischemic traction of the papillary muscles. The incidence of VSDs was significantly higher in the anterior infarct regions than in the inferior/lateral walls (70% vs 29%); however, inferior wall infarctions showed a greater propensity for developing complex VSDs characterized by porous, irregular, or diverse abnormal septal channels.^[3,6]^ The patient in this case study experienced acute inferior MI (right coronary artery occlusion) complicated by VSR at the basal portion of the ventricular septum.

The treatment options can be classified as medication therapy alone, surgical treatment, or interventional closure. Medication therapy alone poses a high risk of early mortality, with 30-day mortality rates reaching 80% in untreated VSDs. Conservative medication therapy is appropriate only for patients with mild hemodynamic compromise or contraindications to surgery.^[7]^ Early surgical correction remains the preferred treatment for AMI complicated by VSR, although optimal surgical timing is still uncertain.^[2]^ Surgical mortality is highest in patients with concurrent cardiogenic shock or those requiring urgent intervention after AMI. Delayed repair of the VSR, defined as surgical intervention occurring days or weeks post-infarction, has garnered attention because of its potential benefits. Studies have suggested that delayed VSR repair lowers mortality rates in patients who develop VSR after AMI. Those undergoing delayed surgery (>7 days post-diagnosis) have demonstrated lower observed mortality, though results may be influenced by patient selection bias and survivor bias.^[2,8,9]^ Some studies indicate that mortality is lowest in patients who undergo surgical repair of VSR on Day 4 post-diagnosis compared to those receiving early intervention.^[10]^ The introduction of temporary mechanical circulatory support devices (e.g., IABP, extracorporeal membrane oxygenation, Impella, and TandemHeart) has fostered a trend toward delayed surgery, with improved survival rates.^[7,11,12]^ In the present case, the patient developed AMI complicated by VSR and exhibited hemodynamic instability, necessitating IABP support and delaying surgery until day 20. IABP counterpulsation support can mitigate left-to-right shunting and enhance hemodynamics in patients with or without shock, enabling them to survive the acute phase and become eligible for surgery, ultimately increasing survival rates.^[2,13,14]^ Percutaneous closure may be considered for patients who are contraindicated for surgery or deemed at extreme risk.^[2]^ The first report of transcatheter closure for post-infarction VSD dates to 1988.^[15]^ Due to the complexity of the procedure, not all patients are suitable candidates, particularly those with posterior wall VSDs, where proximity to the mitral valve and complex local anatomy complicate surgical intervention and can elevate in-hospital mortality.^[1,16,17]^ Outcomes of this procedure compared to surgical treatment remain inconclusive.^[1]^ Some studies indicate no significant difference in long-term post-discharge mortality between patients receiving surgical intervention and those undergoing transcatheter closure.^[18]^ For patients unable to tolerate surgery or transcatheter treatment owing to refractory shock and biventricular failure, long-term mechanical circulatory support, heart transplantation, or total artificial heart implantation may be necessary.^[19]^

## 4. Conclusion

VSR is a rare complication of AMI. Early surgical repair is the preferred treatment. However, the optimal timing for surgery remains uncertain and requires a multidisciplinary and individualized approach. Delayed repair of the VSR may provide certain advantages. For patients with hemodynamic instability, the use of temporary mechanical circulatory support devices to postpone surgery is advisable.

## Acknowledgments

We would like to thank Editage (www.editage.cn) for the English language editing.

## Author contributions

**Conceptualization:** Xuping Cheng.

**Investigation:** Shuang Guo.

**Resources:** Shuang Guo.

**Visualization:** Xuandong Jiang.

**Writing – original draft:** Shuang Guo.

**Writing – review & editing:** Shuang Guo, Xuandong Jiang.

## References

[1] David TE. Post-infarction ventricular septal rupture. Ann Cardiothorac Surg. 2022;11:261–7. 35733715 10.21037/acs-2021-ami-111 PMC9207689

[2] Rao SV O’Donoghue ML Ruel M. ACC/AHA/ACEP/NAEMSP/SCAI guideline for the management of patients with acute coronary syndromes: a report of the American College of Cardiology/American Heart Association joint committee on clinical practice guidelines. Circulation. 2025;151:e771–862. 40014670 10.1161/CIR.0000000000001309

[3] Crenshaw BS Granger CB Birnbaum Y. Risk factors, angiographic patterns, and outcomes in patients with ventricular septal defect complicating acute myocardial infarction. GUSTO-I (global utilization of streptokinase and TPA for occluded coronary arteries) trial investigators. Circulation. 2000;101:27–32. 10618300 10.1161/01.cir.101.1.27

[4] Cubeddu RJ Lorusso R Ronco D Matteucci M Axline MS Moreno PR. Ventricular septal rupture after myocardial infarction: JACC focus seminar 3/5. J Am Coll Cardiol. 2024;83:1886–901. 38719369 10.1016/j.jacc.2024.01.041

[5] Wu J Yan M Chen Y Chen L Hu S. Radiological and hemodynamic parameters in patients with suspected ventricular aneurysm and interventricular septal perforation after acute myocardial infarction: a comparison of non-invasive and invasive diagnostic modalities. Exp Ther Med. 2020;20:961–7. 32742339 10.3892/etm.2020.8754 PMC7388246

[6] Tripathi A Bisht H Arya A. Ventricular septal rupture management in patients with acute myocardial infarction: a review. Cureus. 2023;15:e40390. 37456418 10.7759/cureus.40390 PMC10345166

[7] Arnaoutakis GJ Zhao Y George TJ Sciortino CM McCarthy PM Conte JV. Surgical repair of ventricular septal defect after myocardial infarction: outcomes from the Society of Thoracic Surgeons National Database. Ann Thorac Surg. 2012;94:436–43; discussion 443. 22626761 10.1016/j.athoracsur.2012.04.020 PMC3608099

[8] Arsh H Pahwani R Arif Rasool Chaudhry W. Delayed ventricular septal rupture repair after myocardial infarction: an updated review. Curr Probl Cardiol. 2023;48:101887. 37336311 10.1016/j.cpcardiol.2023.101887

[9] Rashid H Kumar K Ullah A. Delayed ventricular septal rupture repair on patient outcomes after myocardial infarction: a systematic review. Curr Probl Cardiol. 2023;48:101521. 36455796 10.1016/j.cpcardiol.2022.101521

[10] Sánchez Vega JD Alonso Salinas GL Viéitez Florez JM. Optimal surgical timing after post-infarction ventricular septal rupture. Cardiol J. 2022;29:773–81. 35578757 10.5603/CJ.a2022.0035 PMC9550323

[11] Shafiei I Jannati F Jannati M. Optimal time repair of ventricular septal rupture post myocardial infarction. J Saudi Heart Assoc. 2020;32:288–94. 33154931 10.37616/2212-5043.1120 PMC7640570

[12] Liebelt JJ Yang Y DeRose JJ Taub CC. Ventricular septal rupture complicating acute myocardial infarction in the modern era with mechanical circulatory support: a single center observational study. Am J Cardiovasc Dis. 2016;6:10–6. 27073732 PMC4788724

[13] Shi J Levett JY Lv H. Surgical repair of post myocardial infarction ventricular septal defect: a retrospective analysis of a single institution experience. J Cardiothorac Surg. 2023;18:313. 37950265 10.1186/s13019-023-02418-8 PMC10638688

[14] Zhou C Jiang J. Therapeutic efficacy of intra-aortic balloon pump usage in acute myocardial infraction patients complicated with ventricular septal rupture. Chin J Cardiovasc Med. 2021;26:123–6.

[15] Lock JE Block PC McKay RG Baim DS Keane JF. Transcatheter closure of ventricular septal defects. Circulation. 1988;78:361–8. 3396173 10.1161/01.cir.78.2.361

[16] Damluji AA van Diepen S Katz JN.; American Heart Association Council on Clinical Cardiology; Council on Arteriosclerosis, Thrombosis and Vascular Biology; Council on Cardiovascular Surgery and Anesthesia; and Council on Cardiovascular and Stroke Nursing. Mechanical complications of acute myocardial infarction: a scientific statement from the American Heart Association. Circulation. 2021;144:e16–35. 34126755 10.1161/CIR.0000000000000985 PMC9364424

[17] Alfares F Sandhu SK. Postinfarction ventricular septal rupture: transcatheter intervention or surgical repair? J Card Surg. 2021;36:4634–5. 34499380 10.1111/jocs.15987

[18] Giblett JP Matetic A Jenkins D. Post-infarction ventricular septal defect: percutaneous or surgical management in the UK national registry. Eur Heart J. 2022;43:5020–32. 36124729 10.1093/eurheartj/ehac511

[19] Gregoric ID. Total artificial heart in patients with post-infarction ventricular septal defect. Ann Cardiothorac Surg. 2020;9:116–7. 32309161 10.21037/acs.2020.01.04 PMC7160633

